# Primary Hyperaldosteronism as a Cause of Resistant Hypertension: A Case Report

**DOI:** 10.1002/ccr3.73576

**Published:** 2026-09-20

**Authors:** Wasif Majeed Chaudhry, Shazia N. Ibnerasa, Waleed Ahmad, Mohammed Hammad Jaber Amin

**Affiliations:** ^1^ Ghurki Trust Teaching Hospital Lahore Pakistan; ^2^ Lahore Medical and Dental College Lahore Pakistan; ^3^ Alzaiem Alazhari University Khartoum Sudan

**Keywords:** adrenalectomy, case report, hyperaldosteronism, hypokalemia, resistant hypertension, surgery

## Abstract

In young patients with resistant hypertension and hypokalemia, primary hyperaldosteronism should be suspected. Biochemical assessment and adrenal imaging could identify surgically curable aldosterone‐producing adenomas, and a normalization of blood pressure, potassium levels, and symptom resolution can be achieved through adrenalectomy.

## 1 Introduction

The adrenal disorder known as primary hyperaldosteronism (Primary hyperaldosteronism) produces high levels of independently functioning aldosterone which creates an imbalance of electrolytes and blood pressure rise [1]. Dr. Conn identified Primary hyperaldosteronism in 1954 and subsequently defined it through hypokalemia, exceptional potassium urinary excretion, and metabolic alkalosis produced by increased sodium‐retaining hormone which became known as aldosterone [2]. The original report mainly focused on aldosterone‐producing adenoma (Primary hyperaldosteronism); however, medical research now confirms that Primary hyperaldosteronism originates from six distinct adrenal tissues [3].

Most Primary hyperaldosteronism cases (about 90% of cases) include unilateral Primary hyperaldosteronism or bilateral idiopathic adrenal hyperplasia (IHA) whereas less than 10% of Primary hyperaldosteronism cases are categorized under infrequent conditions such as adrenocortical carcinoma and familial hyperaldosteronism. The diagnosis of both classic and subtle Primary hyperaldosteronism forms leads to Primary hyperaldosteronism recognition as the main curable reason for secondary hypertension [3].

Distinguishing between Primary hyperaldosteronism and bilateral adrenal hyperplasia (BAH) is crucial, as their management differs significantly. Primary hyperaldosteronism is primarily treated with adrenalectomy (ADX), whereas BAH is managed with aldosterone antagonists [4]. Diagnostic evaluation uses plasma aldosterone concentration measurement in combination with direct renin concentration (DRC) to generate the aldosterone‐to‐renin ratio (ARR), which represents the most accurate method for Primary hyperaldosteronism diagnosis [5]. Computed tomography (CT) and adrenal vein sampling (AVS) should be utilized to locate the lesion and provide direction for treatments.

In Indonesia, a case series of Conn syndrome was previously reported by Siregar [6], emphasizing the significance of recognizing and managing Primary hyperaldosteronism. We have documented an additional case from a Tertiary care Hopital in Pakistan about patient presentations followed by diagnostic investigations and therapeutic operation alongside histological outcomes. These findings enrich existing research about Primary Hyperaldosteronism management. The SCARE 2025 guidelines have been used to report this case report [7].

## 2 Case History/Examination

A 28‐year‐old female visited the surgical outpatient department (OPD) because she had been dealing with uncontrolled hypertension for four years. The patient is a non‐smoker and a non‐alcoholic drinker. She is a school teacher and stays separately with her family. There is no personal history of any recreational drugs. The patient maintained a healthy status until she started experiencing continuous headaches and vertigo four years ago., prompting a consultation at the internal medicine outpatient department. At that time, her blood pressure was recorded at 170/110 mmHg, and she was started on multiple antihypertensive medications, including Amlodipine/Valsartan (10/160 mg) once daily for one month, Losartan potassium with Hydrochlorothiazide (10 mg) once daily for two months, Amlodipine/Valsartan (5/160 mg) for two months, and a combination of Amlodipine/Valsartan/Hydrochlorothiazide (10/160 mg) for two months. For the last one year, she had been on Amlodipine/Valsartan/Hydrochlorothiazide (10/160/2.5 mg) once daily. Her blood pressure remained out of control even though she received extensive medical treatments while her systolic blood pressure measured between 170 to 180 mmHg and diastolic blood pressure measured between 110 and 120 mmHg. Her complete medical record as well as surgical history showed no abnormalities. The patient had hypertension in their family, but her illness remained under medication control.

The physical assessment revealed a fully responsive patient with a 160‐cm height and 59‐kg weight. The blood pressure recorded in her supine position measured 160/100 mmHg with a heartbeat of 80 beats per minute. Physical examination of the abdomen showed no enlarged organs or visible masses, nor did it detect either striae or abdominal vascular sounds. The patient showed symmetrical peripheral limb pulses and a lack of edema. The patient underwent examinations for cardiovascular, respiratory, and neurological systems without detecting any abnormalities. The patient's routine laboratory check results from blood count to blood glucose and urea and creatinine levels exhibited normal ranges. Urinalysis was also unremarkable.

## 3 Investigations/Differential Diagnosis/Treatment

Electrolyte analysis revealed hypokalemia (2.9 mmol/L). Further biochemical evaluation showed urinary vanillylmandelic acid (VMA) at 18.27 mg/24 h, plasma metanephrine at 10.8 pg/mL, and plasma normetanephrine at 104.73 pg/mL. A 24‐h urine collection measured a volume of 2750 mL. Given the clinical suspicion of primary hyperaldosteronism, additional hormonal studies were performed, revealing a plasma renin level of 5.3 uI U/mL and a serum aldosterone level of 38.20 ng/dL. Biochemical tests excluded Pheochromocytoma and renal causes. Hypokalemia and elevated aldosterone‐renin ratio were in favor of primary hyperaldosteronism diagnosis.

Differential diagnosis was pheochromocytoma, renovascular hypertension, Cushing syndrome, and essential hypertension. Normal plasma metanephrine, normetanephrine, and urinary vanillylmandelic acid eliminated pheochromocytoma. Normal renal functional tests and imaging excluded the renal causes of secondary hypertension. The diagnosis of primary hyperaldosteronism was supported by the presence of hypokalemia and suppressed renin and elevated aldosterone levels.

The diagnostic tests included an ultrasound which showed no abnormalities and an ultrasound.

**FIGURE 1:**
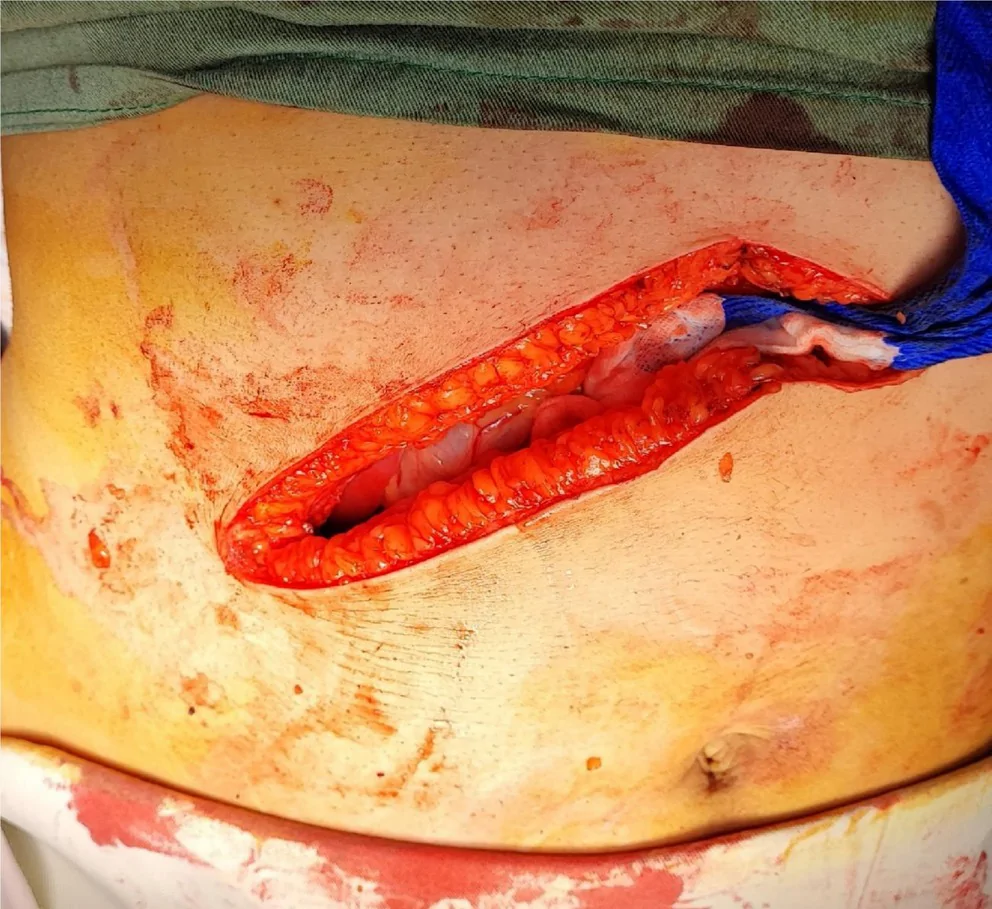
Right subcostal incision for exploratory laparotomy.

**FIGURE 2:**
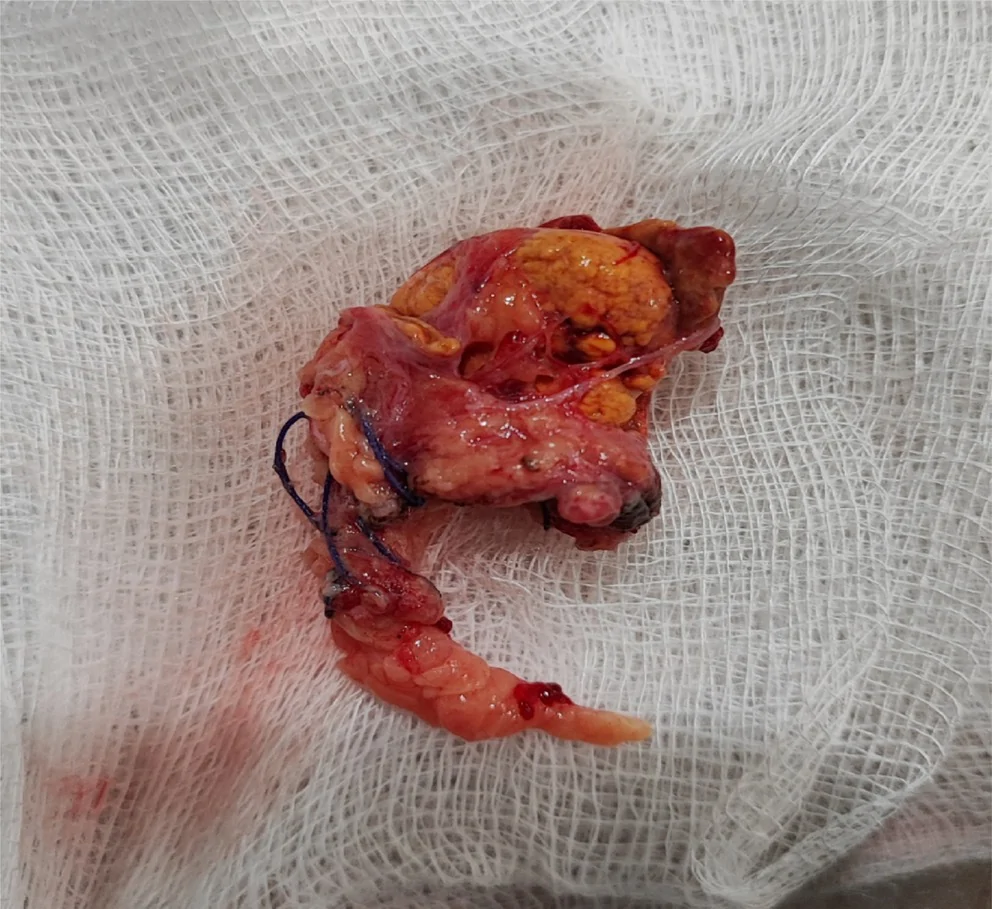
Right adrenal mass after adrenalectomy.

MRI of the abdomen that revealed a well‐defined nodule measuring 17*10 mm in size in the right adrenal gland. Radiological findings combined with clinical indicators led to the establishment of a diagnosis of primary hyperaldosteronism with its origin from a right adrenal mass. Surgical intervention was recommended. TThe preoperative hypertension was under medical control; routine fasting and surgical preparation were applied. Counseling of patients was done and the patient underwent an exploratory laparotomy with right adrenalectomy via a subcostal incision (Figure 1). The adrenal mass was successfully resected (Figure 2), and the patient had an uneventful postoperative recovery. The surgeon was a consultant with adequate experience and the surgery was carried out in a tertiary facility. Perioperative care was consulted with endocrinologists. There were no deviations, and surgery was performed without complications or conversions.

Three days after surgery, the patient remained clinically stable with well‐controlled blood pressure, managed with Nebivolol (5 mg) and Valsartan/Amlodipine/Hydrochlorothiazide (320/10/25 mg). Her serum potassium levels normalized without supplementation. No complications were observed. Prophylactic antibiotic and thrombosis treatment was given. The patient was subsequently discharged in stable condition. Follow‐up and medication were well adhered to by the patient, and there was no side effect of treatment. The Clinical Timeline of the patient is provided in Table 1.

**TABLE 1:** Clinical timeline of the patient.

Date	Event
04/01/2019	Hypertension develops, headache and vertigo develops
04/01/2019–18/01/2023	Several antihypertensive treatments were tried (Amlodipine, Valsartan, HCTZ) and failed to achieve control
14/03/2023	Referral to surgery OPD as a result of amenable hypertension and hypokalemia
24/03/2023	Hypokalemia and increased ratio of aldosterone: renin is revealed following laboratory testing
29/03/2023	MRI reveals right adrenal mass (17 × 10 mm); diagnosis of primary hyperaldosteronism
09/04/2023	Exploratory laparotomy was done and the right adrenalectomy was done
3 Days Post‐op (12/04/2023)	BP returned to normal; serum potassium leveled off without supplementations
3 Months Post‐op (17/07/2023)	Clinical improvement is sustained with continued outpatient follow‐ups with no relapse of symptoms

## 4 Conclusion and Results

The case demonstrates the significance of early diagnosis of primary hyperaldosteronism among young individuals with resistant hypertension and hypokalemia. The proper diagnosis and localization of the condition requires a well‐organized endocrine assessment alongside adrenal imaging. Surgical adrenalectomy is considered the ultimate cure of unilateral adenomas that produce aldosterone, which causes a considerable reduction in blood pressure and electrolyte balance. Indeed, multidisciplinary teamwork is essential to achieve the best diagnosis, management, and postoperative results.

## 5 Discussion

The medical condition known as Primary aldosteronism (Primary hyperaldosteronism) manifests as one documented origin of secondary hypertension that displays elevated aldosterone levels which produce sodium excess along with body fluid increase and decreased renin production. The patient in this case exhibited classic features of Primary hyperaldosteronism, including hypertension and hypokalemia, with high plasma aldosterone concentration (Primary hyperaldosteronism) and reduced plasma renin activity (PRA). The elevated sodium retention that aldosterone regulates leads to high blood pressure and simultaneously produces potassium deficit that causes muscle weakness symptoms together with cramps and paresthesia.

Primary hyperaldosteronism develops most frequently because of aldosterone‐producing adenoma (Primary hyperaldosteronism) or bilateral adrenal hyperplasia (BAH) resulting in 95% of all cases according to [8]. While Primary hyperaldosteronism is responsible for 40% of cases, BAH accounts for 60% [5]. This case involved a right adrenal tumor, which was evaluated using both biochemical and imaging studies to confirm the diagnosis. CT imaging and MRI are extensively used medical imaging techniques that help doctors identify adrenal lesions while establishing the location of these lesions. In this case, MRI revealed an oval, well‐circumscribed right adrenal mass, highly suggestive of an adrenal adenoma. The specificity of unenhanced CT in diagnosing adrenal adenomas is reported to be between 98% and 100%. CT‐based diagnosis of Primary hyperaldosteronism and BAH proves difficult because these two conditions frequently lead to incorrect interpretations in nearly 38% of patients with Primary hyperaldosteronism [9].

The diagnosis of unilateral or bilateral adrenal aldosteronomas requires adrenal venous sampling (AVS) because it provides the most accurate results with a reported sensitivity of 95% and specificity of 100% [10]. AVS helps determine if elevated aldosterone levels stem from one or both adrenal glands so medical professionals can make proper treatment choices. Although this patient had a unilateral adrenal tumor, AVS was necessary to confirm lateralization and avoid misclassification, which could lead to inappropriate treatment.

The treatment of choice for confirmed unilateral Primary hyperaldosteronism needs to be adrenalectomy whereas BAH requires the usage of mineralocorticoid receptor antagonists such as spironolactone [11]. Unilateral adrenalectomy typically results in blood pressure normalization within six months, although in some cases, improvement may take up to a year [8]. Persistent postoperative hypertension is more likely in patients with preoperative hypertension lasting more than six years or requiring multiple antihypertensive medications. Surgical intervention proves advantageous because it manages blood pressure effectively as well as treats organ damage that aldosterone causes independently of hypertension [12]. The prognosis is based on the longevity of hypertension and the age of the patient. Early surgical treatment lowers blood pressure and lowers the risk of cardiovascular diseases.

The diagnostic journey for Primary hyperaldosteronism requires a multi‐step method including biochemical tests and imaging alongside AVS to identify between Primary hyperaldosteronism and BAH. The diagnostic power of imaging methods is limited; hence.

AVS maintains its fundamental role for strategic management decisions. Unilateral Primary hyperaldosteronism patients obtain curative relief through adrenalectomy procedures but bilateral cases need medical treatments instead. The strict adherence to current diagnostic standards enables physicians to deliver precise diagnosis with favorable patient results in Primary hyperaldosteronism cases. There were no serious diagnostic difficulties. AVS was not available but clinical, biochemical, and imaging data demonstrated a confirmed diagnosis. The report is a demonstration of the value of multidisciplinary teamwork and early surgical management in treating primary hyperaldosteronism that led to a successful outcome. One limitation is the exclusion of adrenal venous sampling (AVS) that can be used to establish lateralization in a particular situation. Nevertheless, there was harmony in clinical, biochemical, and imaging reports.

## Author Contributions

**Wasif Majeed Chaudhry:** conceptualization, supervision. **Shazia N. Ibnerasa:** writing – original draft. **Waleed Ahmad:** writing – original draft. **Mohammed Hammad Jaber Amin:** writing – review and editing.

## Funding

The authors have nothing to report.

## Disclosure

*Declaration of AI content*: This case study was not generated by AI tools. While AI was utilized to enhance the professionalism and readability of the content, it was not used extensively to the extent that the work appears AI‐generated. The primary content, analysis, and conclusions are the result of the authors' original work.

## Ethics Statement

The study obtained its ethical authorization from the institutional Ethical Review Committee under reference number 2025/02/R‐04.

## Consent

Written informed consent was obtained from the patient for publication of this case report and any accompanying images.

## Conflicts of Interest

The authors declare no conflicts of interest.

## Data Availability

Within the article, the data used to support the findings of the present study are available. Any additional information is confidential and cannot be disclosed to the audience.
